# The Dresden in vivo OCT dataset for automatic middle ear segmentation

**DOI:** 10.1038/s41597-024-03000-0

**Published:** 2024-02-26

**Authors:** Peng Liu, Svea Steuer, Jonas Golde, Joseph Morgenstern, Yujia Hu, Catherina Schieffer, Steffen Ossmann, Lars Kirsten, Sebastian Bodenstedt, Micha Pfeiffer, Stefanie Speidel, Edmund Koch, Marcus Neudert

**Affiliations:** 1 Department of Otorhinolaryngology Head and Neck Surgery, University Hospital Carl Gustav Carus, TUD Dresden University of Technology, Faculty of Medicine, 01307 Dresden, Germany; 2 Department of Translational Surgical Oncology, National Center for Tumor Diseases (NCT/UCC Dresden), German Cancer Research Center (DKFZ), Helmholtz-Zentrum Dresden-Rossendorf (HZDR), 01307 Dresden, Germany; 3 Else Kröner Fresenius Center, TUD Dresden University of Technology, 01307 Dresden, Germany; 4 Clinical Sensoring and Monitoring, TUD Dresden University of Technology, 01307 Dresden, Germany; 5 Medical Physics and Biomedical Engineering, TUD Dresden University of Technology, 01307 Dresden, Germany; 6 Fraunhofer Institute for Material and Beam Technology IWS, 01277 Dresden, Germany; 7 Ear Research Center Dresden, TUD Dresden University of Technology, 01307 Dresden, Germany

**Keywords:** Translational research, Imaging and sensing

## Abstract

Endoscopic optical coherence tomography (OCT) offers a non-invasive approach to perform the morphological and functional assessment of the middle ear in vivo. However, interpreting such OCT images is challenging and time-consuming due to the shadowing of preceding structures. Deep neural networks have emerged as a promising tool to enhance this process in multiple aspects, including segmentation, classification, and registration. Nevertheless, the scarcity of annotated datasets of OCT middle ear images poses a significant hurdle to the performance of neural networks. We introduce the *Dresden in vivo OCT Dataset of the Middle Ear (DIOME)* featuring 43 OCT volumes from both healthy and pathological middle ears of 29 subjects. DIOME provides semantic segmentations of five crucial anatomical structures (tympanic membrane, malleus, incus, stapes and promontory), and sparse landmarks delineating the salient features of the structures. The availability of these data facilitates the training and evaluation of algorithms regarding various analysis tasks with middle ear OCT images, e.g. diagnostics.

## Background & Summary

The air-filled middle ear cavity consists of the tympanic membrane (TM) and the ossicular chain that connects the TM to the inner ear. Functionally, it matches the impedance of air to the fluid-filled inner ear^1^. The functionality of the middle ear can be disrupted by a variety of conditions such as acute or chronic otitis media or trauma. Pathophysiologically, they result in impaired sound transmission due to perforation of the TM, fixation or disruption of the ossicular chain, or middle ear effusion. Patients perceive this as conductive hearing loss. Current diagnostic modalities, including otoscopy, audiometry and tympanometry, each focus on a single aspect of the pathology. Otoscopy provides a visual assessment of the TM, audiometry evaluates the frequency dependent level of hearing, and tympanometry only assesses the pressure-dependent compliance of the TM.

**Fig. 1:**
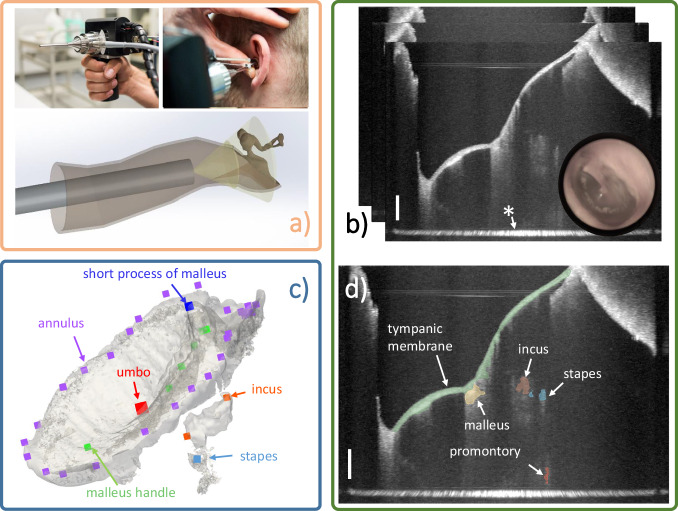
Data acquisition system and data examples. (**a**) Endoscopic OCT device for middle ear data acquisition [adapted from Ref. ^2^, published under CC-BY 4.0 license]. (**b**) Example of B-scans of the acquired image volume combined with the appendant video image. The tympanic membrane is usually fully visible but ossicles including malleus, incus, and stapes are noisy and partially visible, * marks the glass-air interface artifact of the endoscope. (**c**) The translucent 3D model is reconstructed from the OCT segmentation, the points in various colors are the sparse landmarks delineating the salient shape feature of each structure. (**d**) Segmentation example of B-Scan: Tympanic membrane, malleus, incus, stapes, and cochlear promontory are segmented and overlayed to the image slice. Scale bars on the bottom right in (**b**) and (**d**) correspond to 1 mm in air.

As an innovative imaging technology, endoscopic optical coherence tomography (OCT)^2–4^ enables the assessment of both the morphology and function of the middle ear in vivo by the non-invasive acquisition of depth-resolved and high-resolution images. In recent years, several groups therefore developed promising solutions towards in vivo middle ear diagnostics^5–7^. Nevertheless, intrinsic limitations of OCT, e.g. the backscattered light intensity loss over tissue depth as well as the cumulative effect of preceding structures, reduce the signal quality of the target structures, e.g. the stapes, which are further away from the endoscopic probe. Additionally, the OCT volumetric data are usually noisy and often difficult to interpret, especially regarding the identification of deeper middle ear structures such as incus and stapes (see Fig. 1). As a flourishing technique, deep learning facilitates medical image analysis tasks, e.g. segmentation ^8^ and registration ^9^. Thus, the usage of machine learning in the case of middle ear diagnostics is promising, because it has the potential of simplifying the classification of middle ear diseases. Nevertheless, the current bottleneck of the development and application of deep neural networks in the field of middle ear diagnostics is the scarcity of publicly available OCT datasets in this field.

**Fig. 2:**
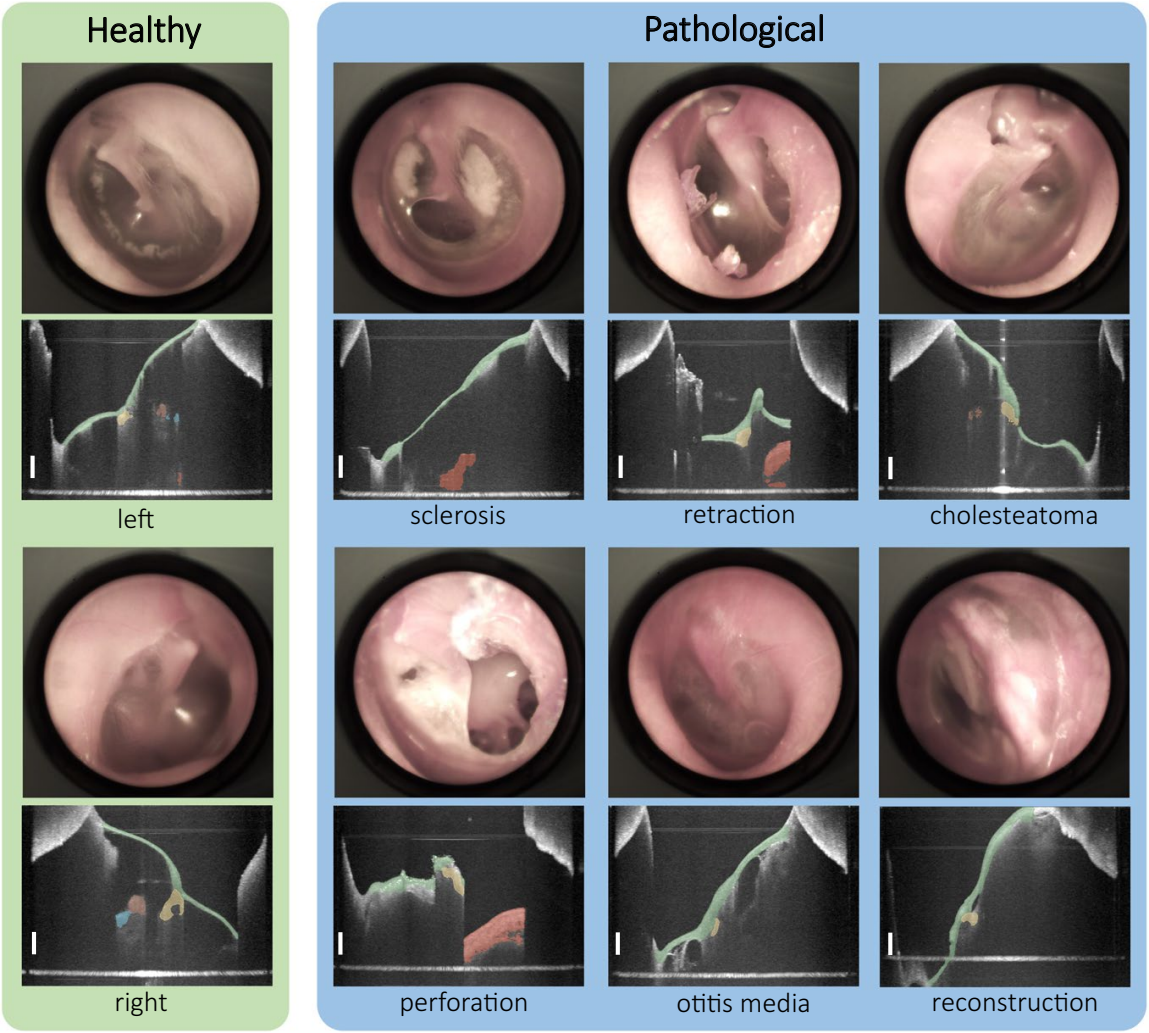
Overview of the comprehensive, segmented OCT dataset and corresponding video images consisting of both healthy and pathological ear data with six different types, including sclerosis, retraction, cholesteatoma, perforation, otitis media and reconstruction. Pathological samples contain an extensive variety of morphology and topology of the middle ear. Scale bars correspond to 1 mm in air.

In this paper, we introduce the Dresden in vivo OCT Dataset of the Middle Ear, which features 43 OCT image volumes from healthy and pathological middle ears of 29 subjects (see Fig. 2). Five essential middle ear structures are segmented including tympanic membrane, malleus, incus, stapes and cochlear promontory. Besides the segmentations, sparse landmarks depicting the salient anatomical features are provided for evaluating algorithms such as detection and registration. Seeing that voxel-wise annotation is a time-consuming task, even for an experienced clinician, we capitalized on the fact that structures like the tympanic membrane are well captured by the OCT volume and the morphological deviation of such structures between healthy ears is slight.

For this, the Human-in-the-Loop^10–12^ (HITL) approach has been proposed and proven to significantly reduce annotation effort while yielding promising results. Following the concept of HITL, we iteratively trained a segmentation neural network namely nnUnet^8^ with an expert correcting the prediction of nnUnet at each iteration. Additionally, we included more pathological samples over the iteration, which vary a lot in morphology and are difficult for the network to segment, so that the learning challenges are reasonably distributed at each step. At the end of the HITL process, the network has been fully trained and is capable of segmenting OCT images from both healthy and pathological middle ears and can be used as a pre-trained model for images from the same and other modalities. In such a way, we alleviated the heavy workload and included more samples with various morphology in the same time scale. The combination of the results from two human raters and the trained neural network rater is checked by the expert and became the final output.

## Methods

This dataset consists of 43 OCT image volumes from both healthy and pathologic middle ears (see Table 1). For each image sample, the semantic segmentation of five anatomical structures including tympanic membrane, malleus, incus, stapes, and promontory is provided. Apart from these, sparse landmarks describing the shape and outline of the segmented structures are marked. Therefrom, sparse point correspondences can be retrieved and contribute to performing or evaluating algorithms of various tasks, e.g. multi-modal image fusion.

**Table 1:** Sample distributions.

	healthy	sclerosis	retraction	cholesteatoma	perforation	otitis media	reconstruction
number of samples	30	1	2	3	2	1	4

Our datasets cover a large variety of middle ear pathology including 6 types.

### Image Acquisition

The OCT volumes were collected between 11.2022 and 08.2023 from clinical daily diagnostics at the University Hospital Carl Gustav Carus Dresden. The subjects consist of two main types: healthy volunteers and patients with age ranging from 22 to 66. This study is covered by the approval of the local Institutional Review Board (IRB00001473) at the TU Dresden (EK 252062017). All patients provided written informed consent to data acquisition, scientific analyses and sharing. Data is anonymized in order to comply with ethical standards and the European Union’s General Data Protection Regulation.

The image acquisition was performed using a custom-built endoscopic OCT system based on the system according to Kirsten *et al*.^2^ (see Fig. 1) with adaptations as described in Golde et al.^13^. A swept-wavelength laser source (SL132120, MEMS-VCSEL, Thorlabs) operates at a sweep rate of 200 kHz, has a center wavelength of 1300 nm, and a wavelength sweep range of 100 nm. In the sample arm’s endoscopic probe, components included are a collimator, two galvanometer scanners for beam guidance, a dichroic mirror for additional visual imaging, and a lens setup featuring GRIN rod lenses. This configuration provides a working distance of 10 mm and an image depth range of approximately 8 mm corresponding to 1024 pixel providing an axial resolution of around 15 *μ*m. With the GRIN endoscope, most of the middle ear is accessed by scanning the proximal surface of the GRIN optics with 500 times 500 A-scans of which approximately 450 A-scans in both lateral directions cover an angular FOV of approximately ± 30°. This spans a field of view (FOV) of around 10 mm at the working distance and thus an approximated lateral resolution of 45 *μ*m. Due to the imaging geometry, the acquired data shows a fan-shape distortion as visualized in Ref. ^4^. Note that, for the sake of preservation of the original information content, the distorted images are stored and act as the target of annotation instead of correcting the fan-shape distortion by geometrically rescaling the volumes using interpolation. Nevertheless, distortion correction can be applied to the data by the provided code such that an isotropic spatial sampling of 20 *μ*m in each direction is obtained.

The measured volumes were processed according to conventional swept-source OCT processing, i.e., background correction, zero-padding, compensating occurring dispersion mismatch, filtering with a Hann window and applying the inverse Fourier transform, using a custom Matlab script (MATLAB R2022b, Mathworks). The acquired volumes were stored in the format of nearly raw raster data (NRRD).

To support the manual image segmentation by less noisy and speckled images, the OCT volumes were additionally processed by applying a tomographic non-local means despeckling (TNode) algorithm by Cuartas-Vélez *et al*.^14^ beforehand. However, the calculation for despeckling is time-consuming and, thus, not suitable for real-time application. Therefore, it was not applied to the data, which were used for the neural network training.

### Segmentation of Anatomical Structures

For semantic segmentation of middle ear structures (tympanic membrane, malleus, incus, stapes and promontory), the open-source software *3D Slicer* (version: 5.2.2, https://www.slicer.org) with the *Segmentation Editor* tool was employed. Three raters including a medical student, a biomedical engineer and an experienced clinician (expert) conducted the segmentation process following the provided segmentation protocol as a guideline (see Supplementary file 1).

In practice, pixel-wise segmentation for all image slices is time-consuming. For each OCT sample, it took at least five hours to segment the volume from scratch including a quality check. Thus, to reduce the workload of such a process, the Human-in-the-Loop approach was harnessed to train a deep neural network (nnUnet^8^) iteratively and to utilize the predictions of nnUnet as pre-segmentations for the other human raters to work on.

**Fig. 3:**
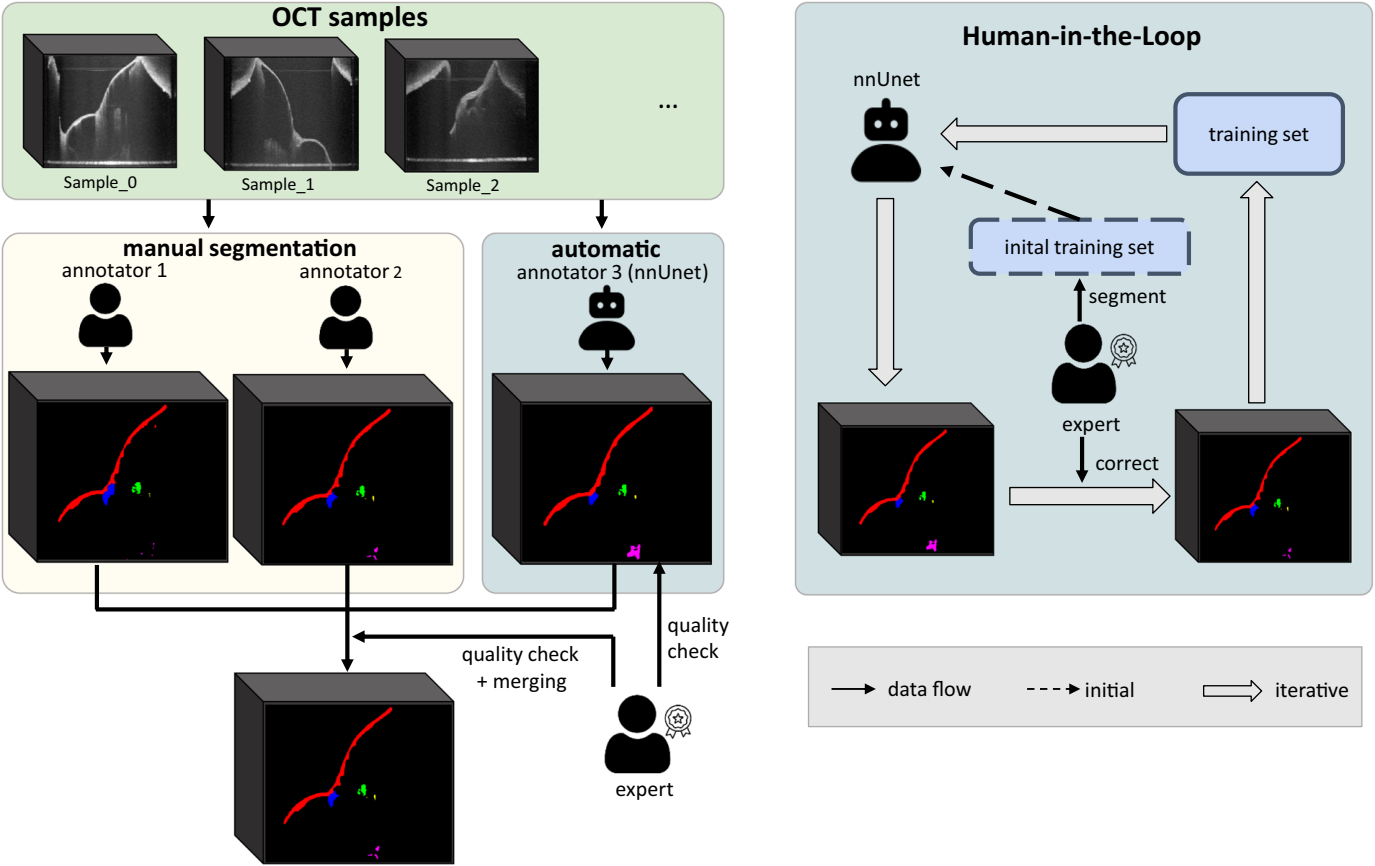
Segmentation scheme. Left: nnUnet is trained following the Human-in-the-Loop strategy and acts as the third rater. The human raters who are an experienced medical student and a biomedical engineer further correct the prediction of nnUnet for the sake of saving effort. The final product is the fusion of the segmentation from three raters and checked by the expert. Right: scheme for Human-in-the-Loop, the expert creates the initial training set to train nnUnet and corrects the inference results on un-segmented samples. The corrected segmentation comprises the new training set for the next iterations.

The HITL procedure is depicted in Fig. 3, which consists of two main phases. In the initial phase, 14 OCT images from healthy ears were segmented manually by an experienced clinician, which comprised the initial training set for nnUnet. Then in the next phase, the clinician corrected the predictions of the network trained from the last phase for unsegmented samples. These new image volumes contained more pathological samples compared to the last iteration. Together with the corrected segmentation masks, they made up the training set for the next iterations. As such, the loop was stopped when the segmentation loss on the test set with 5 samples was low and the prediction was qualitatively approved by the expert. The average time acquired for segmenting each sample was reduced from five hours each to 20 minutes on average. The number of samples for each iteration is listed in the Table 2. Thanks to the HITL process, the two human raters were able to exploit the prediction of nnUnet as pre-segmentation and perform correction until the segmentation accords with real middle ear morphology. At the end of the HITL process, the segmentation results of the latest nnUnet were collected, which is the third rater for all samples. The results were checked by an expert clinician and then merged with the segmentation from two other raters using the *STAPLE*^15^ algorithm. The final segmentation mask was checked by a clinician.

**Table 2:** Samples distribution of HITL iterations.

Sample types	Training			Test	Sum
	1st Iteration	2nd Iteration	3rd Iteration		
Healthy	14 (100.0%)	7 (58.3%)	6 (50.0%)	3	30
Pathological	0 (0.0%)	5 (41.7%)	6 (50.0%)	2	13
Sum	14	12	12	5	**43**

Over the iterations, the percentage of pathological samples, which are difficult to learn compared to the healthy samples, was continuously increased.

### Annotation of Sparse Landmarks

Image segmentation extracts the critical information and simplifies the analysis of OCT images. However, due to the incompleteness of the structures, approaches like multi-modal image fusion can be carried out to reveal the absent parts and further facilitate the interpretation process. Thus, landmarks that describe the morphology of the segmented structures can provide more fine-grained information and act as a performance measurement for the results of fusion algorithms. In this paper, two biomedical data scientists annotated the sparse landmarks (see Fig. 1) of the segmented structures using *3D Slicer* (version 5.2.2) with the *Markup* module. This annotation process is performed under the guidelines which are elaborated in Supplementary file 2. In the last step, the landmarks were checked and corrected by the expert and constituted the final output.

For the tympanic membrane, the *annulus* showing the boundary (about 20 points), and the *umbo*, which is the central point of maximum depression and marks the end of the manubrium, is annotated. Two landmarks are placed to show the *short process of malleus* and the *malleus handle*. *Long process of the incus* is usually partially visible, so corresponding landmark consists of two points, one is the most proximal visible point, and the other one is the distal tip of the long process of the incus, right above the incudostapedial joint. Furthermore, a single point is marked on the *stapes* due to the rare visibility. Note that all these landmarks are marked on the outer side of the epithelium or bones, and are done for the merged segmentation only.

## Data Records

**Fig. 4:**
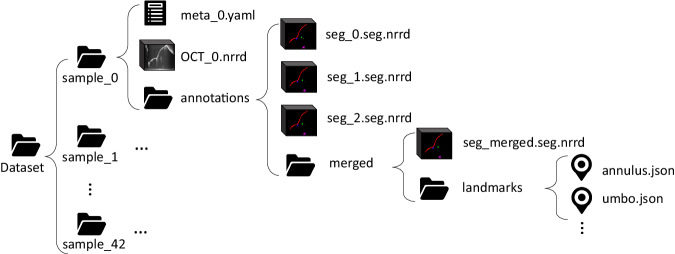
Folder structure of the dataset. From top to lowest level: 1) dataset folder. 2) sample numbers. 3) sample data. 4) segmentation data. 5) merged segmentation and corresponding landmarks. 6) landmarks data.

The DIOME dataset is stored at OpARA (Open Access Repository and Archive, 10.25532/OPARA-279)^16^ and accessible without prior registration. The data folder structure is shown in Fig. 4. 43 sub-folders for 43 OCT middle ear samples compose the first layer. Within each sample folder, three items are listed: a metadata YAML file describing the basic information of the current sample, e.g. if it is a left or right ear, and OCT measurement settings, as well as an OCT image volume in the format of NRRD, and an annotation folder containing all annotation-related items. Within each annotation folder, three NRRD files represent the segmentation results from three raters, and their merged results are saved under the folder next to them. Since the landmarks come along with the merged segmentation, a folder named “landmarks" is placed next to it, which contains six JSON files for the sparse landmarks.

## Technical Validation

To merge the segmentation from the three annotators for each image volume, including two human raters and one segmentation neural network, the *STAPLE*^15^ algorithm was employed. It takes a collection of segmentations of an image and computes simultaneously a probabilistic estimate of the combined segmentation and is often applied in the biomedical field. To validate our segmentations from all three annotators, two metrics commonly used for measuring segmentation performance were calculated:

- **F1 score** is a counting-based metric that measures the overlap between a reference mask and another input segmentation. The value of the F1 score varies from 0 to 1, where 0 means no overlap and 1 full overlap.
- **Hausdorff distance** showcases the maximum distance between two segmentation masks. As a distance-based metric, it usually works as a complement to the counting-based metric and focuses on the assessment of the segmentation boundary and shape. Here we normalized the Hausdorff distance via the diagonal of the 3D image volume. Close to 0 means less separation between the two segmentations, and close to 1 presents larger distance.

The results of the segmentation evaluation are listed in Table 3, where the anatomical structures are ordered based on the distance to the OCT probe. The values in each cell show the average F1 score and Hausdorff distances of all annotators on all anatomical structures. As indicated by comparing the table values, most of the anatomical structures do not have large discrepancies, and the annotators agree with the merged results. However, with the increase in the distance to the probe (from top to bottom), a decreasing tendency of F1 score can be observed which corresponds to the decrease of OCT image quality over depths, e.g. larger noise around the stapes regions. Although the F1 scores of stapes and promontory are lower than other structures, particularly for annotator A1, Hausdorff distances are low enough to prove the rationality.

**Table 3:** Comparison between the segmentation of all raters including two human raters (A1, A2) and one neural network rater (A3).

Structure	A1	A2	A3 (nnUnet)
Tympanic membrane	0.89 (0.034)	0.97 (0.034)	0.90 (0.035)
Malleus	0.80(0.083)	0.82 (0.046)	0.87 (0.137)
Incus	0.85 (0.053)	0.90 (0.038)	0.89 (0.078)
Stapes	0.66 (0.031)	0.77 (0.006)	0.82 (0.025)
Promontory	0.65 (0.018)	0.83 (0.007)	0.78 (0.040)

The F1 Scores and Hausdorff distance in parenthesis are calculated to quantitatively depict the discrepancy between the segmentation results of each rater and the merged ones. A1: medical student, A2: biomedical engineer, A3: neural network (nnUnet).

One interesting fact is the third rater, i.e. the neural network, outperforms the rater A1, the medical student. This proves the comparable capability of the neural network in segmenting OCT images against a human rater.

## Usage Notes

The dataset was published under the Creative Commons Attribution (CC-BY 4.0) license. It can facilitate algorithm development in various deep learning tasks, for example, semantic segmentation, pathology detection or classification, etc. On the one hand, it can be combined with OCT image datasets from other anatomy for fast learning of OCT data and to improve performance. On the other hand, integration with data from other modalities via image registration enables the knowledge transfer to promote the visibility and readability of target structures. For easy processing of the dataset and evaluation of the developed methods, basic functions including 3D model reconstruction, visualization, and metrics calculation are provided.

### Supplementary information

Supplementary_1_How_to_ Segmentation_MiddleEar

Supplementary_2_Annotation_Guideline_for_Markups

## Data Availability

Scripts for segmentation merging and visualization, statistics calculation and fan-shape correction are publicly available at https://gitlab.com/nct_tso_public/diome. All the scripts are written in Python 3.11 and are public under the MIT license.
