# Supplementary material for: The Dresden in vivo OCT dataset for automatic middle ear segmentation
Source: Sci Data. 2024 Feb 26;11:242. doi: 10.1038/s41597-024-03000-0 (PMC10967373; doi:10.1038/s41597-024-03000-0)
Supplement: Supplementary file 1 — Supplementary_1_How_to_ Segmentation_MiddleEar [file 41597_2024_3000_MOESM1_ESM.pdf]

The Dresden in vivo OCT dataset for automatic middle ear segmentation

Supplementary File 1: Guideline for the segmentation of middle ear structures

General:

- the respective structures are visible on every single image of the dataset assigned to them; the degree of recognizability may vary
- avoid marking
  - anything outside the image margins
  - artifacts, i.e. horizontal planes and vertical lines
- mark whenever it is possible to recognize the structure in an area that is
  - small: sometimes, parts of the structure are visible in tiny areas
- note that a structure may be visible in several small areas in a single image

Segmentation steps:

- Select a segment, e.g., *Tympanic membrane,* by clicking on it
- Choose the visibility of the segments by turning on/ off the “eye” left of the segment name
- At the beginning, use *Threshold* tool, adjust to the general brightness and contrast by using the lower slider button and press A*pply* to finish it
- Continue with segmenting using tools from the tool bar:
  - Islands
    - Remove small islands after using the threshold tool to remove artifacts
  - Paint/Erase
    - Manual marking in every single slice
    - May set edible intensity range
    - Use sphere brush only when marking clear separated structures
  - *Scissor*
    - *Works in 3D view*
    - *Delete bigger parts of segments*
    - *add parts of certain segments to other segments*
  - *Fill between slices*
    - create new segment for it
    - mark slices with paint tool
    - applied for all visible segments
  - *smoothing*
- try not to delete clear visible structures → put it in the segment *Misc*
- Yellow arrow under tool bar is *undo*

Tympanic membrane:

- Cone-shaped, often diagonal in the image
- Connected to ear canal wall
- Be careful in areas of overframing, especially in areas parallel to the reference plane

Malleus:

- Start from the umbo, try to follow structure
- Surrounded by tympanic membrane tissue
- Often in the middle of the image

- Compare orientation to VIS camera image

Incus:
- Located in the upper posterior quadrant
- Oriented from 11 to 5 in right ears, from 1 to 7 in left ears

Stapes:
- Located below the long process of the incus
- rarely visible
- orient in the 3D view

Promontory:
- usually at the lower bound of the image
- often overlayed by the artifact
- often low contrast
