# Supplementary material for: The Dresden in vivo OCT dataset for automatic middle ear segmentation
Source: Sci Data. 2024 Feb 26;11:242. doi: 10.1038/s41597-024-03000-0 (PMC10967373; doi:10.1038/s41597-024-03000-0)
Supplement: Supplementary file 2 — Supplementary_2_Annotation_Guideline_for_Markups [file 41597_2024_3000_MOESM2_ESM.pdf]

The Dresden in vivo OCT dataset for automatic middle ear segmentation

Supplementary File 2: Guideline for annotation of sparse landmarks of anatomical structures of the middle ear

General:

- mark the points that can show the most salient feature of the anatomical structures
- use the 3D view for assistance to orientate oneself
- have the data volume and the segmentation open
- place the points at the surface

Tympanic membrane:

1. Annulus:
    - delineate the border of the tympanic membrane as closed curve
    - start from the pars flaccida area, but do not include it
    - artifacts need to be avoided, e.g., specks
2. Umbo:
    - deepest point of cone
    - the center point of maximum depression
    - on the outer side of the tympanic membrane

Malleus:

1. Short process of malleus
    - outer most point
2. malleus handle
    - curve along in 3D (hide tympanic membrane) from umbo to the short process of the malleus after having it orientated as you would look on to it
    - border to TM
    - at least five points due to the curvature of the malleus handle

Incus:

1. Long Process (proximal → distal)
    - only part of the incus handle is visible
    - nearly parallel to the malleus handle
    - at least two points

Stapes:

- try to mark the most lateral point
- usually only part of the stapes head is visible
- artifacts and noise
